# RETRACTION: Aggravation of Alzheimer's Disease due to the COX‐2‐Mediated Reciprocal Regulation of IL‐1β and Aβ Between Glial and Neuron Cells

**DOI:** 10.1111/acel.70158

**Published:** 2025-07-08

**RETRACTION**: P. Wang, P.‐P. Guan, T. Wang, X. Yu, J.‐J. Guo and Z.‐Y. Wang, “Aggravation of Alzheimer's Disease due to the COX‐2‐Mediated Reciprocal Regulation of IL‐1β and Aβ Between Glial and Neuron Cells,” *Aging Cell* 13, no. 4 (2014): 605‐615, https://doi.org/10.1111/acel.12209.

The above article, published online on 13 March 2014 in Wiley Online Library (wileyonlinelibrary.com), has been retracted by agreement between the journal Editor‐in‐Chief, Monty Montano; the Anatomical Society; and John Wiley & Sons Ltd. The retraction has been agreed upon following an investigation based on concerns raised by a third party. Duplications were uncovered between the NF‐κB bands presented in Figure 2C and the β‐actin bands in Figure 4K. Further duplication between the β‐actin bands in Figures 4E and 5B, 4I and 5A, and between 4J and 5A were also observed. The authors collaborated with our investigation, but due to the time that has elapsed since publication, they were unable to produce any original data. As a result, the editors have lost confidence in the results and conclusions presented in this study. The authors did not confirm agreement with the final wording of the retraction.

